# Supplementary material for: Identification of Triploid Plants in Seed-Derived Progeny of Cultivated Olive
Source: Plants (Basel). 2026 Jan 1;15(1):127. doi: 10.3390/plants15010127 (PMC12787707; doi:10.3390/plants15010127)
Supplement: Supplementary file 1 [file plants-15-00127-s001.zip › plants-4069944-supplementary/Figure S1.pdf]

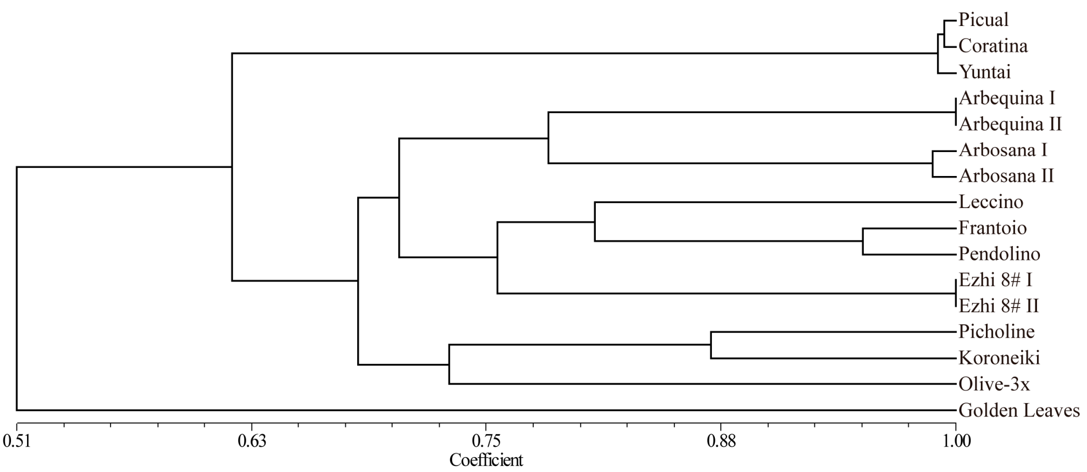

Figure S1. Dendrogram of 16 olive genotypes generated using 64 polymorphic InDel markers in NTsys 2.10e based on Euclidean distance. The clustering pattern shows that the triploid 'Olive-3x' groups closely with 'Koroneiki', supporting the marker-based inference of their genetic relationship.
