# Supplementary material for: Identification of Triploid Plants in Seed-Derived Progeny of Cultivated Olive
Source: Plants (Basel). 2026 Jan 1;15(1):127. doi: 10.3390/plants15010127 (PMC12787707; doi:10.3390/plants15010127)
Supplement: Supplementary file 1 [file plants-15-00127-s001.zip › plants-4069944-supplementary/Table S1.pdf]

**Table S1.** Primer sequences for the 64 InDel markers developed in olive.

| Marker code | Forward primer | Reverse primer |
|---|---|---|
| OE01-1 | TCACCCGAAAGAAGAAGCAA | AGGAACAAACAATGCAGAGGT |
| OE01-4 | GACTTAAGCATGGCCACAGG | CCAACTCAGTCTGGGATCTTGT |
| OE01-10 | CCTTTGGTGGCTGCAATTCT | TGGGTTTGAAAGTGGCAATGA |
| OE01-15 | CAGCGTTGGTAGAGTGGAGG | CCACGCACACACAGTTGC |
| OE02-1 | TCACGTGTAGTTGCATGCTT | AGCGGCAGAGAAGATTTCAGT |
| OE02-5 | TGGACCCAAGACAACGATCT | ACATATTGGTCGGCTCCTTCA |
| OE02-9 | AGGGCCCTTCATTGCTCATT | AGCTTCCGATCTTTTCCTGCA |
| OE02-16 | TCCCCTCTTCTCAAAATGGCC | TGGAAGCCGGTTTATCGATCT |
| OE02-20 | TGTCACAACTCTACCTCCAGC | GGCAAAACAACCACCGTGAA |
| OE03-7 | GTTGGCCTTCTGACTCCTCA | AGCCACAACTTGCCTGATCT |
| OE03-11 | AACTCCGGTGACCTTTCCTG | AGGCTCAAGGATCTGTTCACA |
| OE03-16 | ACACTTCTCTTTGACCGGTGA | TCCTGTTGTAGCTAACCTCTGT |
| OE03-17 | TGCTTTAGAGGACCTTGCGA | GCAAGGATTATTCTACCCAGCA |
| OE03-18 | GACTCAAACAAGTGCGCCAA | ACATAATATGCTGCCGGACAC |
| OE04-2 | TTTGCCCACTCACCAATCCA | TGGTAGCAGCATCATCGACA |
| OE04-19 | TTCGGGAACATTTGCACCAA | GTGGTTGACATGCTCACTTCA |
| OE04-20 | AATTTGGTGGAATCGCGTCG | GGACCAAGATATGCCACTGAGA |
| OE05-12 | ACACAGTGGCACCAAGAGAA | AAATTGCGGCTCACTCCTGA |
| OE06-14 | AGACATTGGCTTGGCATCAA | CCAAAGGTAGTAGACGGTGCT |
| OE06-16 | TCGATCCAAAGCCAATCCGA | AGGCCTTCTGATATGCTGCA |
| OE06-19 | AGGAACCTGTCACCTTTCATGT | CTCCCAAAATCACGAACGCA |
| OE07-7 | ACAAATGGTCCTCGGGGTAT | AGATCATCTCAAGGGCCTTCC |
| OE08-11 | AGCCAGCTCTAGTTTCAACCT | ACGCTAAGTTCATAGGTCTGGA |
| OE08-15 | TATGCTCACCGTTCTGCTGA | GACGAGAGATAGGAAGGCACA |
| OE09-1 | TCTAGAACCTCGAGAGCTGG | ACGAACCTTTGATGATTGGGTG |
| OE09-6 | TGAGGATCCCGATAAGTGCA | AGAGCCTTTCGAAATGTCTCCA |
| OE09-10 | GGTAGTTGCCATGATCTGATGA | TGGCCACAAATCACCACAAA |
| OE10-3 | TCTTGGAGGACACAGAGACA | AGAGAGAAACATTTTAGCCGGT |
| OE10-7 | TCTCACACATTCTTGAAGCCCA | ATAACCCCGCTTGCAAACAA |
| OE11-5 | CTCCACGCTTTTGTAATTCGC | ACCAGTTCCCAATGCTTTCA |
| OE11-7 | TGAACTACACCTGGGCAGAA | CAGCACTTTGTTCATCGACGA |
| OE11-9 | ACACATGTCGATTCCAAGGAGA | TCACGGAGACTCTATGAAAGGA |
| OE11-18 | AATTGTACACGGGGCAAAGG | AGAAGTCACTCATCCTGCTCA |
| OE12-16 | TGATCATCTCACCATCCACCT | AGCCCAATTTCCTCACCCAT |
| OE12-19 | GCACTCAGATTACAACAAGCCT | GCGAACATCAGAGAGAGAGAGA |
| OE13-3 | TGACAGAGATTTGGATGGTGGA | GAGATTCTGTGTGTGTGTGTGA |
| OE13-4 | ACGTCACTCCATCTTCTCTCT | TCGTGCTAGGTGGAATTGGT |
| OE13-14 | TGATTGTTCCGACTCTGAGCA | TGTGTGTGAGGAGCTAAGGT |
| OE13-16 | TGCCTCGTTGAATTCTCCCC | TCAGCGCCCTCAATCACAAT |
| OE14-4 | TGAGAAGGAGAGGGAGAGTGA | CCAACGGGTCCAAAAGTTCA |
| OE14-10 | AAGGCAGCTGGTGTAAGAAG | ACCTCGCTATGCTTGTATGGT |
| OE14-11 | GTGGTGGGGCTATGTTAACC | TGTTCCGTTTGCAAGTGGAT |

| | | |
|---|---|---|
| OE14-13 | TGGTGCAAAACGGAGAACAT | TGGACATGTGAATGCCTGAA |
| OE15-2 | TGCTTAGAAATGAGTCCGACAC | ATTCACCTCAGCCCACAATG |
| OE15-4 | AAATTCGAGCAGGGCCTTTT | CCGGTTCAAGTTGGAGGAAGA |
| OE15-10 | ACCATCCTACAGAAATTGCCT | AGTTGAGGGACCTATGCTCA |
| OE16-1 | GGTAATTGGGCTGAACGAAACC | CGCAGAGAAGAAAACCGACG |
| OE16-12 | ACCAGCAGCAGAATCAAACAC | TCCCATCTTTGAATTCTTGCCA |
| OE16-17 | TCTGCTTCTCCATAATCCCCT | AGATACGTTAGATTGTTGGCCT |
| OE17-5 | TATTCCAGGCCCAGAGTCCA | GGTAACAACACTGAAACACACG |
| OE17-7 | GGAAAGCAGTCAACTACAGGC | TGTAGGGATTCGACTGTCAGT |
| OE17-18 | TGCGCAGTTCATAGATTAGACA | TAGAGTTGGTTGAGTGCAGC |
| OE18-7 | ACCTCAACATGTTACAGGCA | TCATGTGGGACGATACTCATCT |
| OE18-16 | TGTTCTATTCATCTGCTGCGT | GAGAAGAACAGTGAACTCCTCA |
| OE18-20 | CAACACGGATACATGAACCCA | TGAAGGTGTTGAGACTAGCCA |
| OE19-2 | AGTTTCGATTCCAGGCTCCA | CCTCCTCTTCAGTTATTCGACC |
| OE19-3 | GCAAGACAAACGGGTGAACA | GCTTATGCCTCGAATCTTGTCT |
| OE19-9 | TTGGAACGTGCATTTCTGGA | TTGGAACGTGCATTTCTGGA |
| OE20-19 | TGACAACAAAAGCCGGATGT | TCTTTGCTTGAGTCACATTCCT |
| OE21-13 | TCCCAAAAGTTAGCCAGTGAG | AGCTACTGTACTGAGGTTCGT |
| OE22-9 | GCCCAGATTCGTAAAGCCAA | TGGCTCACTTCACCTCTTGT |
| OE22-16 | TCTTGAATGAACCACACTCCA | TGGAGTGAAGGAACATTGCC |
| OE22-17 | CCAAACATGGGTCGATTAGGT | TCGAATTAAATGTCCCCGAGGT |
| OE23-20 | ACGCTTATTGATAGGGGACACA | AATTCACAAGCTGGCACACC |
